# Management of pancreatic and duodenal trauma in childhood: a university hospital experience over a 10-year period

**DOI:** 10.1007/s00068-024-02506-x

**Published:** 2024-03-26

**Authors:** Agah Koray Mansiroglu, Emrullah Cesur, Binali Firinci, Ozgur Caglar, Murat Yigiter, Ahmet Bedii Salman

**Affiliations:** 1 Pediatric Surgery, Ministry of Health Sivas State Hospital, Örtülüpınar, Yeni Bulv. No:55, 58040 Sivas, Turkey; 2 Pediatric Surgery, Ministry of Health Amasya Sabuncuoğlu Şerefeddin Education and Research Hospital, Amasya, Turkey; 3 Pediatric Surgery, Ataturk University Research Hospital, Erzurum, Turkey; 4 Pediatric Surgery, Ankara Etlik City Hospital, Ankara, Turkey

**Keywords:** Duodenal injury, Pancreatic injury, Pediatric trauma

## Abstract

**Purpose:**

Duodenal/pancreatic injuries occur in less than 10% of intra-abdominal injuries in pediatric blunt trauma. Isolated duodenal/pancreatic injuries occur in two-thirds of cases, while combined injuries occur in the remaining. This study aimed to investigate pediatric patients with pancreatic and duodenal trauma.

**Methods:**

Data from 31 patients admitted to Atatürk University, Medical Faculty, Department of Pediatric Surgery for pancreatic/duodenal trauma between 2010 and 2019 were retrospectively analyzed. Age/gender, province of origin, duration before hospital admission, trauma type, injured organs, injury severity, diagnostic and therapeutic modalities, complications, hospitalization duration, blood transfusion requirement, and mortality rate were recorded.

**Results:**

Twenty-four patients were male, and 7 were female. The mean age was 9 years. The leading cause was bicycle accidents, with 12 cases, followed by traffic accidents/bumps, with 7 cases each. Comorbid organ injuries accompanied 18 cases. Duodenal trauma was most commonly accompanied by liver injuries (4/8), whereas pancreatic injury by pulmonary injuries (7/23). Serum amylase at initial hospital presentation was elevated in 83.9% of the patients. Thirty patients underwent abdominal CT, and FAST was performed in 20. While 54.8% of the patients were conservatively managed, 45.2% underwent surgery.

**Conclusion:**

Because of the anatomical proximity of the pancreas and the duodenum, both organs should be considered being co-affected by a localized trauma. Radiologic confirmation of perforation in duodenal trauma and an intra-abdominal pancreatic pseudocyst in pancreatic trauma are the most critical surgical indications of pancreaticoduodenal trauma. Conservative management’s success is increased in the absence of duodenal perforation and cases of non-symptomatic pancreatic pseudocyst.

## Introduction

Trauma is the most common pediatric mortality cause, with a rate of 49% [1]. Abdominal trauma is children’s second most common trauma site, following isolated head trauma [2, 3]. Duodenal and pancreatic injuries have been reported to occur in 2–12% of children suffering from abdominal injuries [4, 5]. Isolated duodenal and pancreatic injuries have been observed in approximately two-thirds of cases, while combined injuries in both organs have occurred in the remaining third [6]. The most common cause of pancreatic and duodenal injuries in pediatric patients worldwide has been reported as falling from a height [7]. On the contrary, in a 17-year clinical experience of pancreatic trauma published by Ravi Kumar Garg and Jai Kumar Mahajan in 2017, it was reported that the most common cause of pancreatic injury was bicycle handlebar injury [8]. Additionally, motor vehicle accidents, assaults (such as punches and kicks), falls from bicycles, collisions, child abuse, endoscopic biopsy procedures, foreign body ingestions, and penetrating traumas at an ever-increasing rate constitute other causes of pancreatic and duodenal trauma [9–15].

Because pancreatic and duodenal injuries are rare traumas, the follow-up of these traumas is controversial in the literature. Pediatric pancreatic and duodenal injury management is mainly based on adult data, although there is a steadily growing body of research on this topic. Surgical intervention was not required in most pediatric patients admitted to the hospital with a history of blunt trauma [16]. Many pediatric centers have reported surgery rates of 1% or less; however, postoperative meticulous care and follow-up have become necessary in these patients to achieve a low mortality rate in patients requiring surgery [17, 18].

We aimed to retrospectively review our pediatric patients hospitalized for pancreatic and duodenal trauma regarding their diagnostic characteristics and therapeutic management to improve the outcomes of these rare and complex injuries.

## Material and methods

Following approval of the Ethics Committee of Atatürk University Medical Faculty with decision # B.30.2.ATA.0.01.00/147, data from 31 patients admitted to Atatürk University, Faculty of Medicine, Department of Pediatric Surgery for pancreatic and/or duodenal trauma between 2010 and 2019 were retrospectively analyzed. Age, gender, province of origin, duration before admission to the hospital, type of trauma, injured organs, injury severity, diagnostic procedures, treatment modalities, complications, length of hospitalization, blood transfusion requirement, and mortality rate were recorded. Data acquisition and evaluation were performed on a computer using IBM SPSS Statistics software.

## Results

### Patients’ general demographics

Of the 31 patients who were followed up and treated in our clinic for traumatic pancreatic and/or duodenal injuries, 18 patients from Erzurum were admitted to our clinic, and 13 were admitted from neighboring cities. Seven were female, and 24 were male. The patients’ mean age was 9 (0–17) years. The patient age group distribution is presented in Table 1.

**Table 1:** Distribution of demographic, clinical, laboratory, and prognostic data of our pediatric pancreatic/duodenal trauma patient series

	Pancreatic (*n* = 23)	Duodenal (*n* = 8)	Total (*n* = 31)
Demographics			
Gender (*n*)			
Male	21	3	24
Female	2	5	7
Age (years) (*n*)			
0–5	4	1	5
6–11	10	3	13
12–17	9	4	13
Trauma type (n)			
Falling from height	4	-	4
Traffic accident	4	3	7
Collision	6	1	7
Falling from bicycle	8	4	12
Gunshot injury	1	-	1
Accompanying injury sites (n)			
Liver	-	4	4
Lung	7	2	9
Skeleton	2	2	4
Spleen	4	-	4
Kidney	1	-	1
Stomach	1	-	1
Laboratory results at admission			
Serum amylase (mean ± SD)	527.00 ± 513.70	155.87 ± 79.12	
Serum AST (mean ± SD)	74.36 ± 92.56	161.13 ± 138.49	
Serum ALT (mean ± SD)	49.05 ± 91.36	126.00 ± 108.22	
Hemoglobin (gr/dl) (mean ± SD)	12.5 ± 2.36	12.7 ± 1.36	
Injury grading (n)			
I	13	2	15
II	1	5	6
III	9	1	10
IV	-	-	-
V	-	-	-
Treatment strategy (n)			
Conservative	16	1	17
Octreotide	4	-	4
Erythrocyte suspension	3	1	4
Surgery	7	7	14
Nutritional status			
TPN (*n*)	7	3	10
Duration of oral feeding restriction (days) (mean)	8.5	10.6	
Duration of nasogastric decompression (days)	7.5	9.25	
Clinical course/outcome			
Duration of hospitalization (days) (mean)	19.57	18	
Intensive care follow-up	6	3	9
Mortality	0	0	0

### Trauma types experienced by our pediatric patients

The most common cause of injury was falling from a bicycle, with 12 cases, followed by traffic accidents and collisions, with 7 cases each (Table 1). Handlebar marks were detected on physical examination in 5 of the 12 patients who presented to the hospital after bicycle accidents.

### Accompanying injury sites

Concomitant organ injuries accompanied pancreatic/duodenal trauma in eighteen cases. While liver injury was the most prevalent comorbidity of duodenal trauma, pancreatic trauma was most commonly accompanied by lung injury. The distribution of concomitant organ/system injuries is shown in Table 1.

### Duodenal and pancreatic injury sites

Anatomically, the most common duodenal injury site was the second part of the duodenum (*n* = 4), followed by the first part (*n* = 3) and the third part (*n* = 1). The pancreatic body (*n* = 9) and the pancreatic tail (*n* = 9) were the most prevalent pancreatic injury sites, followed by the pancreatic head (*n* = 5). In four cases, both pancreas and duodenum injuries were simultaneously observed. Among these, two cases were included in the duodenal injury group because the primary injury was to the duodenum, whereas the other two cases were assigned to the pancreatic injury group since the primary injury was to the pancreas.

### Diagnostic stage

#### Laboratory results

Serum amylase, AST, ALT, and hemoglobin levels were measured at admission in all pancreatic and/or duodenal trauma patients. The mean serum amylase, AST, and ALT values in the case group were significantly high (Table 1). On the other hand, serum amylase values at the time of hospital admission were within the normal range in 3 patients with pancreatic trauma and 2 with duodenal trauma. When the mean serum amylase values obtained at initial admission to the hospital were analyzed, it was noted that the mean serum amylase value of the Grade I pancreatic trauma case group was lower than those of the higher-grade patient groups (Table 2). However, a statistical conclusion could not be achieved because the number of cases was small. A similar suggestion is valid for duodenal trauma even though differences among the grades were present.

**Table 2:** Number of pancreatic/duodenal trauma cases by grade, mean discharge times, mean serum amylase levels at initial presentation, and mean duration of amylase level elevation

	Average duration to discharge (days)	Mean amylase level at admission (U/dl)	Mean duration of amylase level elevation (days)
*Pancreatic injury grade (n)*			
Grade I (13)	13.6	457.7	34.8
Grade II (1)	14	1368	14
Grade III (9)	28.6	533.6	84.7
Pseudocyst (11)	30	673.2	85.2
*Duodenal injury grade (n)*			
Grade I (2)	16	181	6.5
Grade II (5)	15.2	146.4	3.2
Grade III (1)	36	296	30

#### Imaging results

An upright postero-anterior abdominal radiogram was obtained in all patients. Twenty patients underwent FAST, and 30 patients underwent abdominal tomography. When the admission abdominal radiographs of the patients treated for duodenal injury were analyzed, perforation was found in only one patient. In addition, extravasation was found in one patient, free air under the diaphragm in two patients, and duodenal hematoma in one patient in passage radiographs obtained during follow-up.

**Fig. 1:**
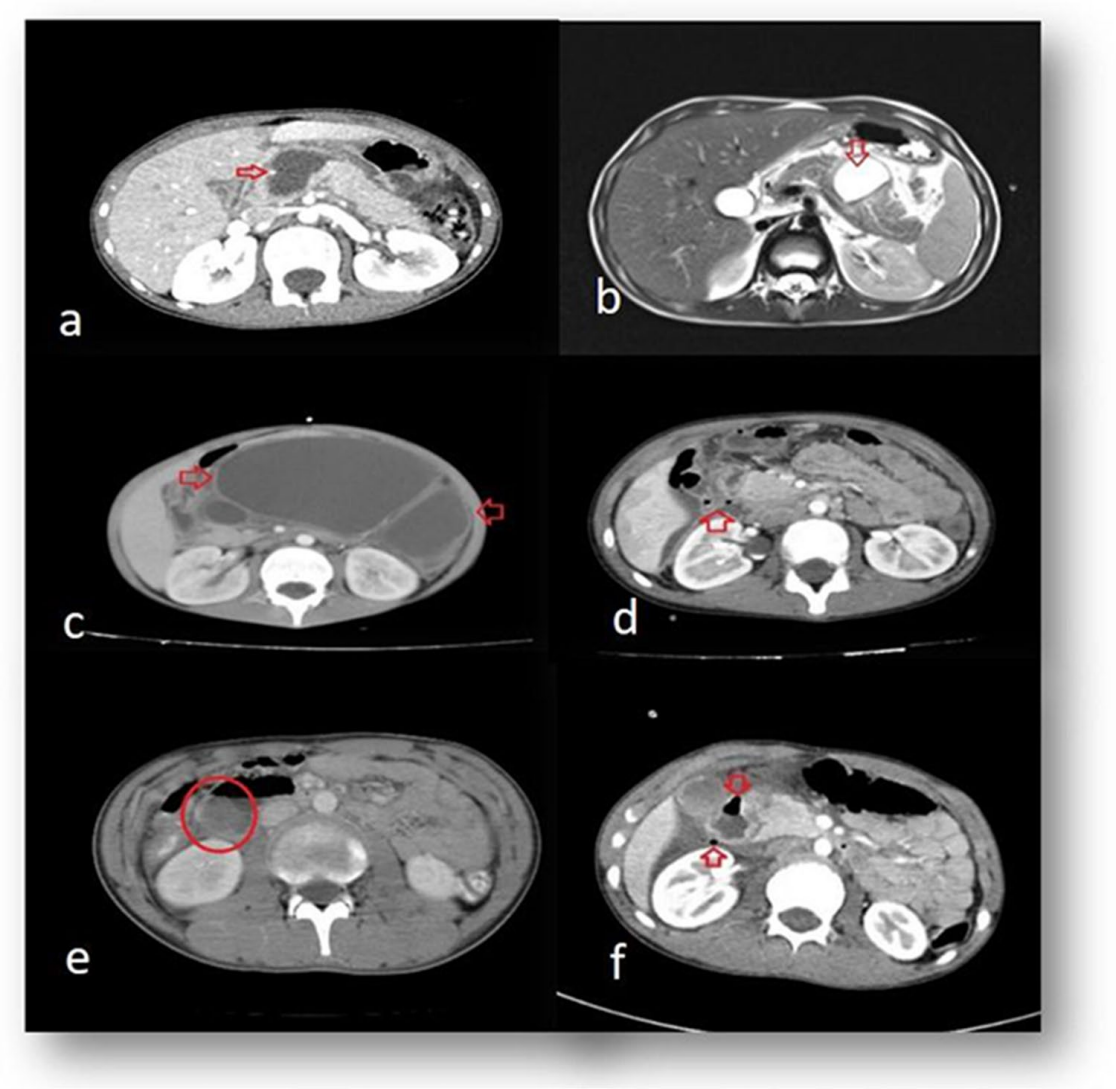
Various CT and MRI images of the pancreatic/duodenal trauma cases. **a** Pancreatic laceration on abdominal CT (arrow); **b** pancreatic laceration on abdominal MRI (arrow); **c** pseudocyst image on abdominal CT (arrows);** d** image of free air belonging to duodenal perforation on abdominal CT (arrow); **e** duodenal hematoma on abdominal CT (circle);** f** impaired duodenal wall integrity and free air on abdominal CT (arrows)

**Fig. 2:**
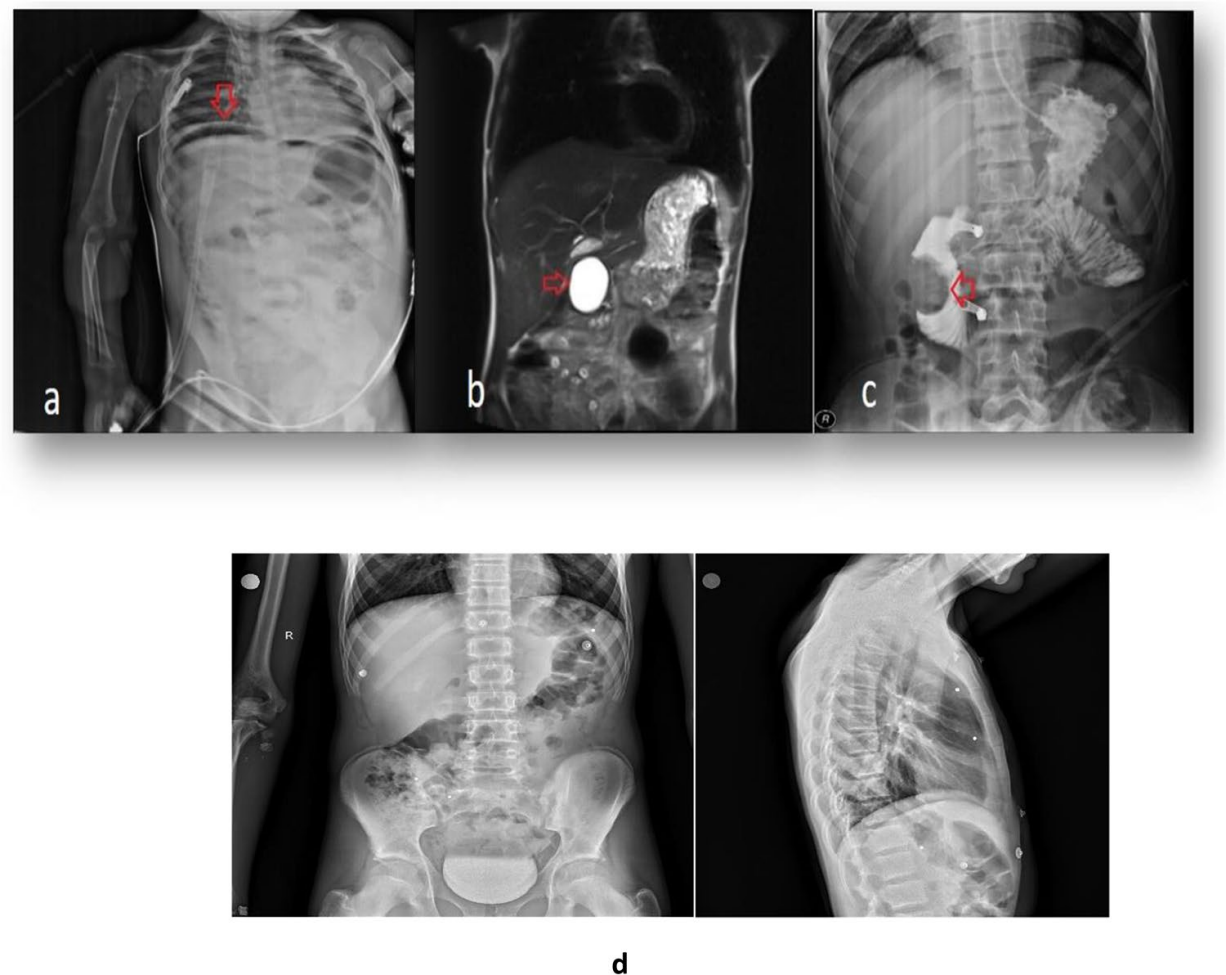
Various radiological images of pancreaticoduodenal trauma cases. **a** Duodenal perforation, free air under the right diaphragm on a direct radiograph (arrow). **b** The pseudocyst’s image on abdominal MRI (arrow). **c** Duodenal hematoma filling the lumen on gastric-duodenal contrast radiography (arrow). **d** Direct anteroposterior and lateral X-ray images of the patient with pancreatic trauma due to gunshot injury (radiopaque pellets are observed)

FAST was performed in 15 patients with pancreatic trauma at admission to the hospital. All 23 patients with pancreatic trauma underwent abdominal CT at admission, whereas FAST was performed in 5, and abdominal CT was performed in 7 duodenal trauma patients. Five of 8 duodenal trauma patients and 3 of 23 pancreatic trauma patients underwent passage radiography due to suspicion of intestinal perforation and absence of clinical improvement. Abdominal MRI was used in five patients with pancreatic trauma during their treatment, and a pancreatic pseudocyst was detected in three of them. Various direct X-ray, CT, and MRI images of the pancreatic/duodenal trauma cases are presented in Figs. 1 and 2.

**Fig. 3:**
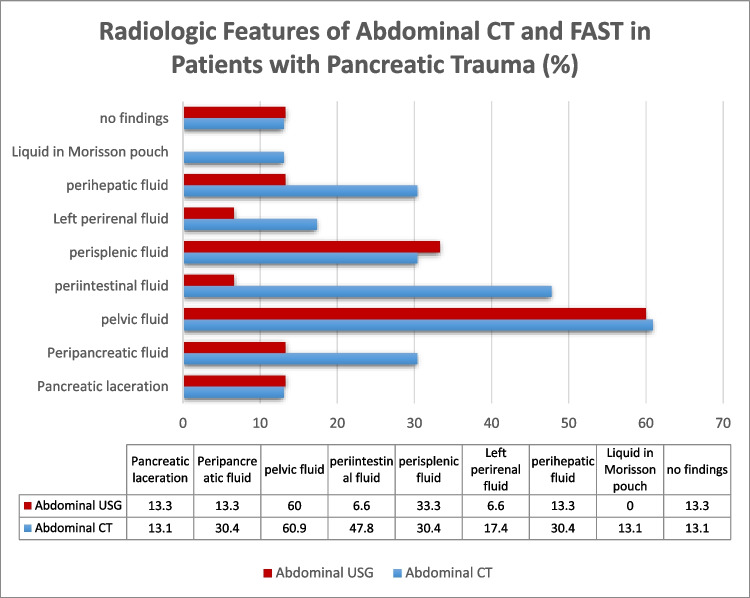
Radiologic features of abdominal CT and FAST in pancreatic trauma patients

**Fig. 4:**
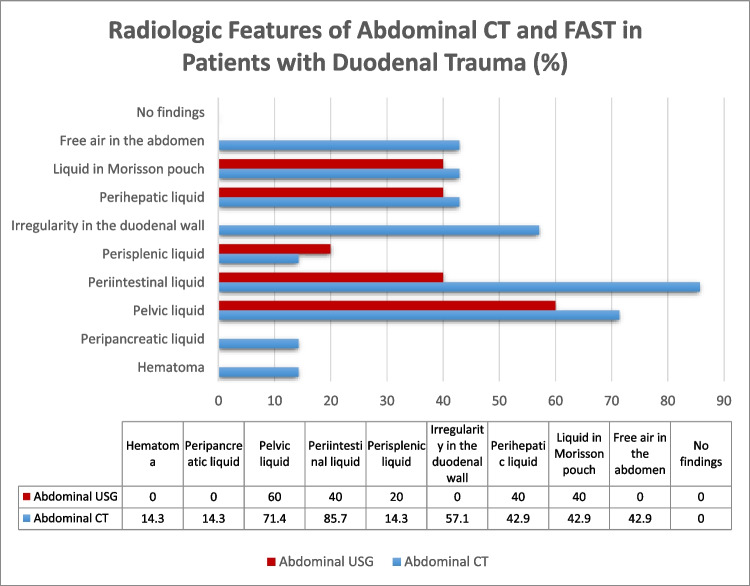
Radiologic features of abdominal CT and FAST in duodenal trauma patients

Abdominal CT was superior to FAST in diagnosing or raising suspicion of pancreaticoduodenal traumas when the assessments performed at admission were analyzed (Figs. 3 and 4). An endoscopy was performed on one patient due to accompanying upper GI bleeding; however, the endoscopy revealed normal findings.

In pancreatic trauma, diagnosis was made at the time of admission in 21 of 23 cases, whereas within 24 h of admission in one patient, the duration was unidentified in one patient. The diagnosis of duodenal trauma was made within 24 h in four patients, within 48 h in three patients, and within 1 week in one patient (delayed presentation). Overall, the diagnostic process of duodenal trauma was delayed by an average of 2.1 days from the moment of trauma.

### Therapeutic management

#### Pancreatic trauma

Of the 23 patients who were followed up for pancreatic trauma, 7 were operated on. Of the 13 Grade I pancreatic traumas, one case underwent percutaneous drainage because of a subsequently developing pseudocyst, and another case was diagnosed during laparotomy for gunshot injury and followed up with drain placement. One Grade II case was treated conservatively. Four of the nine Grade III cases were successfully managed conservatively, whereas five patients underwent percutaneous cyst drainage. Two of these patients benefited from the procedure, whereas a Roux-Y cystojejunostomy procedure was performed in one patient who did not benefit from percutaneous cyst drainage. The open surgical procedure achieved cyst drainage and necrotic pancreatic tissue debridement in the remaining two cases. Drains were placed in all patients who underwent surgical procedures.

#### Duodenal trauma – surgery

Among 8 duodenal trauma patients, seven were diagnosed with duodenal perforation and operated on. Four patients underwent primary repair + omentopexy only, whereas one underwent primary repair + omentopexy and gastrostomy, one underwent duodenoduodenostomy, and one underwent duodenoduodenostomy with gastrojejunostomy. Drains were placed in all patients who underwent surgical procedures.

#### Duodenal trauma – conservative

One patient was diagnosed with duodenal hematoma and was treated conservatively; no operation was planned since the size of the duodenal hematoma did not increase, flow on gastric decompression was reduced, and the patient tolerated nutrition. In the other two Grade 1 duodenal injuries accompanying pancreatic injury, duodenal wall abnormalities were detected and treated conservatively.

### Clinical course and outcome

Serum amylase levels were monitored during inpatient treatment and after the patients were discharged. There was no bile leakage in the follow-up of the anastomosis and primary repairs with a drain inserted, but two patients developed brid ileus after discharge. All patients were given prophylactic antibiotics starting from admission. While hospitalized with duodenal trauma, one patient developed candida sepsis, and another developed a fever secondary to pleural effusion.

Long-term follow-up of patients with pancreatic/duodenal trauma was performed with nasogastric drainage. Especially in duodenal trauma, patients were not fed for a long time, and anastomosis lines were not strained with the help of TPN; thus, TPN was started in ten patients to help avoid potential surgical complications. Four patients required blood transfusions (Table 1).

### Pseudocyst development

Pseudocysts developed in 11 patients with pancreatic trauma, six of them necessitating surgical interventions. The timing of pseudocyst surgery was 2, 11, 22, 23, and 60 days post-admission in five of them, respectively. The sixth patient’s surgery timing data was unreachable. One patient who had undergone percutaneous cyst drainage experienced re-development of pseudocyst.

### Octreotide use in pseudocyst development

Regarding octreotide use in pseudocyst, four of the patients with pancreatic trauma were started on octreotide, a somatostatin analog, and the common features of the patients were the presence of a concomitant pseudocyst, prolonged elevated amylase levels for more than 30 days, and prolonged hospitalization. Two of these three Grade III patients underwent surgery, and the amount of postoperative drainage decreased with octreotide use. In the other patient with Grade III pancreatic trauma who was started on octreotide, the pseudocyst diameter decreased from 8 to 3 cm. Likewise, the pseudocyst diameter of the Grade I pancreatic trauma patient started on octreotide decreased.

The mean discharge time was 19.57 (2–123) days in pancreatic trauma patients, whereas 18 (7–35) days in duodenal trauma patients. All patients were discharged after the termination of their treatments (Table 1).

## Discussion

The epidemiology of pediatric pancreaticoduodenal trauma has not been well-studied. However, some data suggest that it accounts for less than 1% of all pediatric traumas and 2–12% of abdominal traumas in children [4, 5]. In our study, trauma was more prevalent in male patients. The fact that more than half of the cases were admitted from the province of our hospital may be because the relevant clinics manage lower-grade traumas deemed not to necessitate surgery in the neighboring provinces. While trauma-related admissions within the province occur more rapidly, transportation problems in the surrounding provinces might also have affected this situation. Lack of available trauma centers in the surrounding provinces to follow up multi-trauma, the inadequacy of intensive care units that can provide treatment in cases of need, the fact that specialist physicians working in the surrounding provinces have not seen sufficient cases of duodenal and pancreatic trauma in the institutions where they received training, and even if they have, the scarcity of the number of cases constitute the most important reasons for referral.

It is known that patients with Grade IV and V trauma are exposed to higher-energy traumas, and vital organ injuries are more prevalent in them. Disruptions in the transportation network and problems during patient transport are also associated with these traumas. For these reasons, patients with Grade IV and V pancreatic and/or duodenal traumas, the incidence of which is typically low, could not be followed up and treated in our clinic.

The most common cause of pancreatic and duodenal injuries in pediatric patients has been reported to be falling from a height [7], whereas, in our clinic, it was falling from a bicycle. While the bicycle handlebar was the cause of trauma in 28% of bicycle injuries worldwide, this rate was found to be almost half in our clinic. In our study, falling from a height was the fourth most common cause. Blunt trauma is the most common underlying mechanism of pediatric pancreatic and duodenal injuries [3]. In our clinic also, we observed that most pancreatic and duodenal injuries were due to blunt trauma. While the literature has reported that the coexistence rate of pancreatic and duodenal traumas is approximately 33.3% [6], this rate was low in our study.

In the study published by Adamson WT et al. in 2003, abdominal CT was performed in all trauma patients with an elevated amylase level, and pancreatic laceration was detected in 51% of their abdominal CTs [19]. In our study, 20 of 23 patients with pancreatic trauma had elevated serum amylase levels at initial hospital presentation. Even though Grade I pancreatic injury was encountered in two of eight duodenal trauma patients, amylase elevation was observed in six patients with duodenal trauma. When both of our results were analyzed, we concluded that although elevated serum amylase level is an essential marker for pancreatic trauma, it should be kept in mind that its elevation is also present in duodenal trauma cases.

The diagnosis rate of intestinal perforations developing after abdominal trauma using direct radiography was reported as 52% [20]. Due to the duodenum’s anatomical location, the diagnosis rate by direct radiography at the moment of admission in our clinic was low. This rate increased three-fold on the subsequent contrast-enhanced abdominal radiographs. While serial abdominal CT scans have been recommended to prevent diagnostic delay, we showed in our clinic that serial contrast-enhanced direct abdominal radiographs could also avoid diagnostic delay, and thus, patients were less exposed to radiation.

Regarding speed and radiation exposure, FAST is more advantageous than tomography. It can be performed at the bedside, even in unstable patients. In duodenal and pancreatic injuries, the evaluation of lesions by physicians with insufficient FAST experience raises concerns about the reliability of the FAST among physicians. As found worldwide and in our study, FAST has lower sensitivity and specificity than tomography in pancreatic and duodenal trauma [21].

The fact that abdominal CT is less invasive, more accessible, and more widely applicable than peritoneal lavage has narrowed the scope of diagnostic peritoneal lavage. The difficulty of laparoscopic surgical intervention after diagnostic laparoscopy of the duodenum and pancreas, which are located retroperitoneally and deeply, has prevented diagnostic laparoscopy from being a favored diagnostic method [22].

Englum et al. gathered data from trauma centers in the USA and showed that the proportion of conservative treatment for pancreatic injuries was 76.3% [3]. This percentage was 69.6% in our study, similar to Englum et al.’s result. Moreover, in our study, duodenal traumas were found to be diagnosed later than pancreatic traumas, whereas the mean discharge time of pancreatic trauma patients was longer than that of duodenal trauma patients. This was due to the conservative treatment being preferred to surgical treatment primarily for pancreatic trauma, the waiting time for intervention in pseudocyst developing later, and the fact that surgical indication margins were not as precise as in duodenal trauma.

## Conclusion

Because of the anatomical neighborhood of the pancreas, a solid organ, and the duodenum, a luminal organ, the possibility of both organs being affected by a trauma to that region should always be considered. It is important to consider the possibility of duodenal injury when high amylase levels are detected after abdominal trauma. Duodenal injuries (perforation/hematoma) that are not visible on initial abdominal tomography can be detected with less radiation through contrast-enhanced abdominal radiography. Positive physical examination findings, persistent clinical symptoms accompanying radiologic evidence of perforation in duodenal trauma, and an intra-abdominal pancreatic pseudocyst in pancreatic trauma have been recognized as the most critical surgical indications regarding pancreaticoduodenal trauma. A high success rate of conservative treatment in pancreatic and/or duodenal trauma was associated with the absence of duodenal perforation and regression of clinical symptoms of pancreatic pseudocyst with supportive treatment.

## Data Availability

The authors would provide the data if requested.
